# Imaging Mass Spectrometry Technology and Application on Ganglioside Study; Visualization of Age-Dependent Accumulation of C20-Ganglioside Molecular Species in the Mouse Hippocampus

**DOI:** 10.1371/journal.pone.0003232

**Published:** 2008-09-18

**Authors:** Yuki Sugiura, Shuichi Shimma, Yoshiyuki Konishi, Maki K. Yamada, Mitsutoshi Setou

**Affiliations:** 1 Department of Bioscience and Biotechnology, Tokyo Institute of Technology, Yokohama, Kanagawa, Japan; 2 Mitsubishi Kagaku Institute of Life Sciences, Machida, Tokyo, Japan; 3 National Institute for Physiological Sciences, Okazaki, Aichi, Japan; 4 Hamamatsu Medical School, Department of Molecular Anatomy, Hamamatsu, Shizuoka, Japan; Instituto de Tecnologia Química e Biológica, Portugal

## Abstract

Gangliosides are particularly abundant in the central nervous system (CNS) and thought to play important roles in memory formation, neuritogenesis, synaptic transmission, and other neural functions. Although several molecular species of gangliosides have been characterized and their individual functions elucidated, their differential distribution in the CNS are not well understood. In particular, whether the different molecular species show different distribution patterns in the brain remains unclear. We report the distinct and characteristic distributions of ganglioside molecular species, as revealed by imaging mass spectrometry (IMS). This technique can discriminate the molecular species, raised from both oligosaccharide and ceramide structure by determining the difference of the mass-to-charge ratio, and structural analysis by tandem mass spectrometry. Gangliosides in the CNS are characterized by the structure of the long-chain base (LCB) in the ceramide moiety. The LCB of the main ganglioside species has either 18 or 20 carbons (i.e., C18- or C20-sphingosine); we found that these 2 types of gangliosides are differentially distributed in the mouse brain. While the C18-species was widely distributed throughout the frontal brain, the C20-species selectively localized along the entorhinal-hippocampus projections, especially in the molecular layer (ML) of the dentate gyrus (DG). We revealed development- and aging-related accumulation of the C-20 species in the ML-DG. Thus it is possible to consider that this brain-region specific regulation of LCB chain length is particularly important for the distinct function in cells of CNS.

## Introduction

Gangliosides are glycosphingolipids consisting of mono- to poly-sialylated oligosaccharide chains of variable lengths attached to a ceramide unit. They are inserted in the outer layer of the plasma membrane with the hydrophobic ceramide moiety acting as an anchor, while their oligosaccharide moiety is exposed to the external medium[1]. Gangliosides are particularly abundant in the central nervous system (CNS) and are thought to play roles in memory formation[2], neuritogenesis[3], synaptic transmission[4], and other neural functions. In addition, they are particularly involved in brain development and maturation[5], [6].

**Figure 1:**
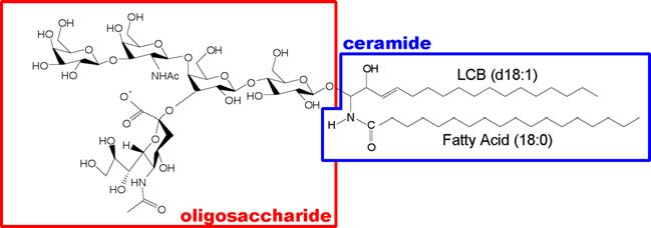
Structure of C18-LCB containing GM1a. Gangliosides comprise a large family; their oligosaccharides structures differ in the glycosidic linkage position, sugar configuration, and the contents of neutral sugars and sialic- acid content. The ceramide moiety of gangliosides, it also has some variation varies with respect to the type of long -chain base (LCB) (sphingosine- base) and fatty acid moiety.

Gangliosides comprise a large family (Figure 1); their constituent oligosaccharides differ in the glycosidic linkage position, sugar configuration, and the contents of neutral sugars and sialic acid. Based on the number of sialic-acid contained, they subdivided into GM (i.e. mono-sialilated), GD (di-sialilated), GT (tri-sialilated) and GQ(quadra-sialilated) groups. The oligosaccharide unit is important because gangliosides interact with proteins that participate in signal transduction through membrane microdomains. For example, the ganglioside GM3 has been found to be closely associated with signaling proteins, such as c-Src, Rho, FAK, and Ras, in cultured cells[3], [7], [8], and GD3 is associated with the Src-family kinase Lyn and the neural cell adhesion molecule TAG-1 in rat brain[9], [10].

The ceramide moiety of gangliosides also varies with respect to the type of long-chain base (LCB) (sphingosine base) and fatty acid moiety. Such structural heterogeneity results in part from the different chain lengths, especially of the LCB (See also Figure S1). While some complex mammalian sphingolipids such as C18-sphingosine, i.e., C18-LCB species, are distributed in all tissues, C20-sphingosine (C20-LCB species) is present in significant amounts only in the gangliosides of the nervous system[11]–[14], and its content increases throughout life[15]–[17]. This structural heterogeneity of ceramides allows flexibility for performing different cellular functions, for example, cAMP-mediated signal transduction[18]. Thus, it has been suggested that C18- and C20-gangliosides are differentially regulated and might play different roles in neuronal function *in vivo* [11].

At present, few methods exist for the holistic study of the distribution of these ganglioside molecular species in biological specimens. Antibodies to some oligosaccharide moieties are available for visualizing the molecular species with different constituent oligosaccharides[19], but immunological methods cannot detect the differences in the ceramide structure, which is hidden in the lipid bilayer.

In this respect, IMS of biological tissues by using matrix-assisted laser desorption/ionization (MALDI) is a useful method. It can distinguish between different ganglioside molecular species by determining the differences in the mass-to-charge ratios (*m/z*) simultaneously[20]–[24]. Furthermore, use of tandem mass spectrometry (MS^n^) to examine tissue surfaces enables identification of the visualized molecules and further provides detailed information on their structures[21], [25]–[27].

In this study, we used IMS to perform molecular imaging of ganglioside molecular species in mouse hippocampal formation. We clarified the distributions of different ganglioside molecular species, especially of those that contain different LCB moieties, namely C18- and C20-sphingosine. We have demonstrated, for the first time, that the distribution of ganglioside molecular species *in vivo* is brain-region selective. We speculate that this selectivity is associated with the different functions of the gangliosides expressed in different brain regions.

## Results

### Detection of gangliosides directly from mouse hippocampal formation

**Figure 2:**
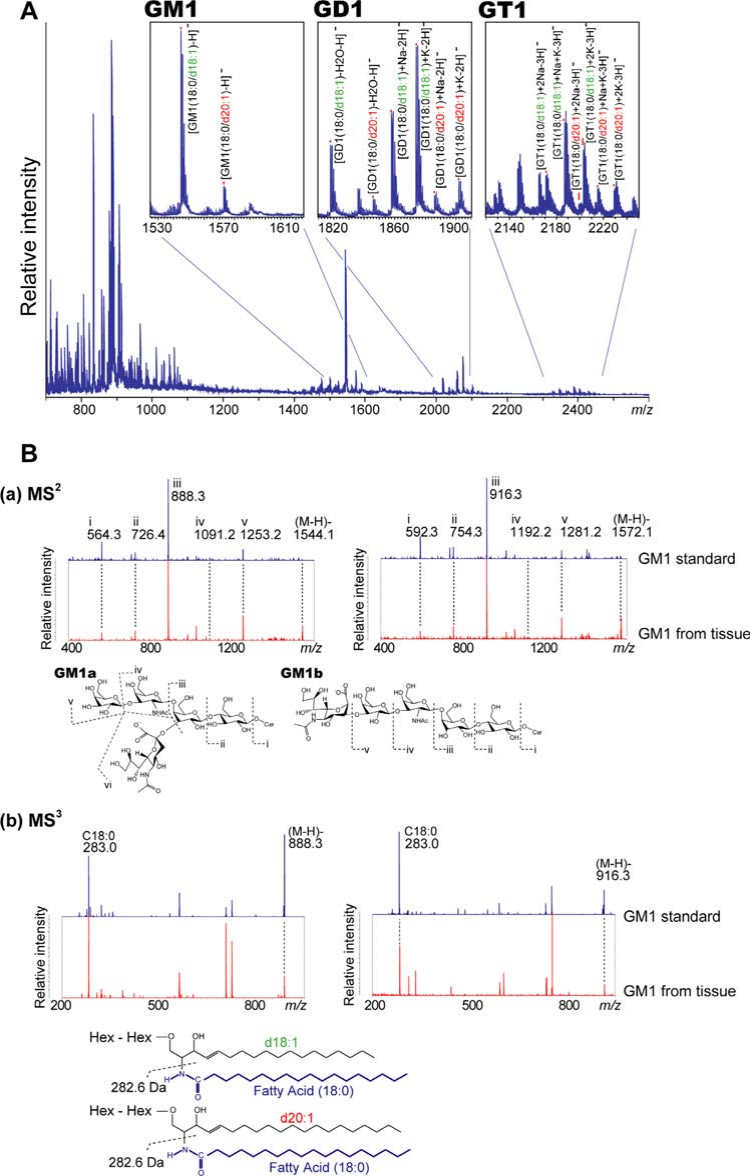
Direct MALDI-MS and MS^n^ allows specific detection of ganglioside molecular species. A. Averaged mass spectra obtained from the entire hippocampal formation. In the spectra, the mass peaks corresponding to GM1, GD1, and GT1 are detected, and IMS provides distinct signals for molecular species containing C18- and C20-sphingosines. B. MS^n^ structural analysis of ions corresponding to GM1. (a) MS^2^ product ion spectra show that the ions at *m*/*z* 1544 and 1572 had the same oligosaccharide structure, i.e., they contained a sialic acid moiety, but the ceramide mass peaks were observed at different *m*/*z* values. (b) MS^3^ product ion mass spectra of *m*/*z* 888.3 and 916.3 were obtained to determine the different structural constituents in the ceramide moieties. Because of the detection of *m*/*z* 283.0 (fatty acid-related ion) in both the spectra, the 28-u difference between *m*/*z* 1544 and *m*/*z* 1572 was attributed to the difference in the sphingosine constituent; *m*/*z* 1544 had C18-sphingosine and *m/z* 1572 had C20-sphingosine.

The negative-mode MALDI-MS spectra obtained directly from the mouse hippocampal formation are shown in Figure 2A. Negatively charged glycerophospholipids, such as phosphatidyl inositol, phosphatidyl ethanolamine, and phosphatidyl serine, and sphingolipids such as sulfatides (STs) were detected in the mass range of 800<*m/z*<950. Mass peaks corresponding to GM1, GD1, and GT1 gangliosides were detected in the mass range of 1500<*m/z*<2300. As shown in Table 1, we detected ions corresponding to GM1, GD1, and GT1, which contain either C18- or C20-sphingosine.

**Table 1:** 

	Negative Ions					
	[M-H]^−^	[M+Na-2H]^−^	[M+K-2H]^−^	[M+2Na-3H]^−^	[M+Na+K-3H]^−^	[M+2K-3H]^−^
GM1 (d18:1/18:0)	1544	-	-	-	-	-
GM1 (d20:1/18:0)	1572	-	-	-	-	-
GD1 (d18:1/18:0)	-	1858	1874	-	-	-
GD1 (d20:1/18:0)	-	1886	1902	-	-	-
GT1 (d18:1/18:0)	-	-	-	2170	2186	2202
GT1 (d20:1/18:0)	-	-	-	2198	2214	2230

Structural analysis by MS^n^ allows us to analyze more detailed structure of detected ions. To confirm that the differences of 28-u which corresponds to a (CH_2_)_2_ unit, observed between the C18 and C20 species, can be certainly attributed to the LCB chain lengths, we performed a structural analysis of ions corresponding to GM1 gangliosides by MS^n^ (Figure 2B). MS^n^ can provide detailed structural information of the ions of interest. The MS^2^ results for both *m/z* 1544 and 1572 showed a ceramide peak and peaks corresponding to oligosaccharides containing a sialic acid (Figure 2B (a)). The peaks in the MS^2^ spectra for oligosaccharides of *m/z* 1544 and 1572 were exactly the same; thus, these gangliosides have the same oligosaccharide moiety. We therefore performed MS^3^ of the ceramide peak to determine the detailed structure of the ceramide. In the MS^3^ spectra obtained, the common peak was observed at *m/z* 283.0, which corresponded to (C_17_H_35_COOH)^−^, a fatty acid (Figure 2B (b)). Thus, we determined that the mass difference was derived from the difference in the chain lengths of the LCB, namely C18- and C20-sphingosine.

### IMS of gangliosides in the mouse hippocampal formation

**Figure 3:**
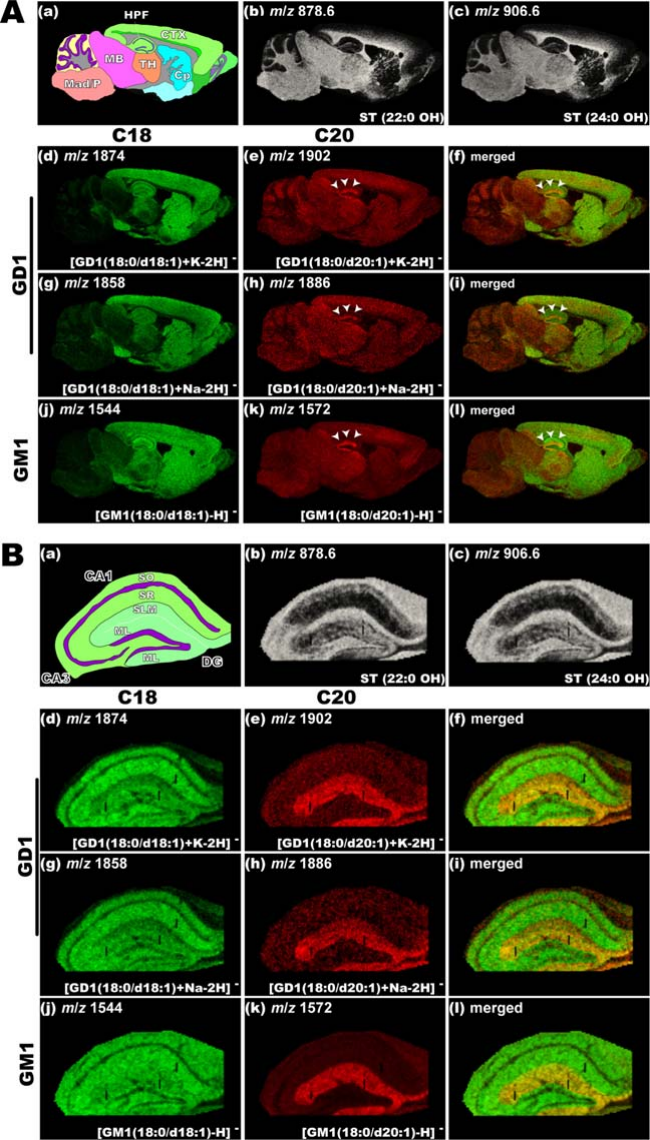
Localization of C20-sphingosine-containing gangliosides in the hippocampal formation. IMS at 50 µm raster step size was used to gain an overview of ganglioside distribution in different brain regions (A), and IMS at 15 µm raster size was used to study in detail the distribution pattern of gangliosides in the hippocampus (B). In both panels, schematic diagram of the brain section (a) and ion images of STs (b–c) are presented. For ions corresponding to the GD1 molecular species, we observed the ion distributions of both sodium and potassium complexes, i.e., the ions at *m/z* 1858 (f) and *m/z* 1886 (g), which correspond to the [M+Na-H]^−^ form of C18- and C20-GD1, and those at *m/z* 1874 (h) and *m/z* 1902 (i), which correspond to the [M+K-H]^−^ form of C18- and C20-GD1, respectively. The ion distribution patterns corresponding to the GD1-Na salts and GD1-K salts are fairly uniform for both C18- and C20- species. For GM1, *m/z* 1544 (d) and *m/z* 1572 (e), which correspond to C18- and C20-sphingosines containing GM1 respectively are shown.

MALDI-IMS visualizes the spatial abundance of numerous ions simultaneously in the same tissue section, thus enabling holistic imaging of ganglioside molecular species. Figure 3 shows the imaging results obtained for the mouse sagittal brain section at low instrumental step size (50 µm raster). For imaging the myelinated region of the brain section, we visualized the ions at *m/z* 878.6 and 906.6, which correspond to STs with different sphingosine bases, namely ST(22:0 OH) and ST(24:0 OH), respectively (Figure 3 b–c). STs demonstrate the same distribution pattern regardless of the type of ceramide moiety; however, interestingly, the distributions of C18- and C20-ganglioside molecular species are considerably different. In particular, IMS revealed a characteristic concentration of ions corresponding to C20 species of both GM1 and GD1 in a part of the hippocampal formation (Figure 3A, arrowheads). On the other hand, ions corresponding to C18-GD-1 were distributed uniformly in the gray matter region of the frontal brain, and those corresponding to C18-GM1 were strongly distributed in the white matter region in addition to the gray matter region (Figure 3A).

To understand the characteristic localization of the C20-species in the hippocampal formation in greater detail, we performed IMS of the hippocampal formation at high instrumental step size (15 µm raster) (Figure 3B). Ions corresponding to the C20-species of both GM1 and GD1 were found to be localized in the outer two-thirds of the dentate gyrus (DG) molecular layer (ML) and the stratum lacunosum moleculare (SLM) of both CA1 and CA3. They were, however, much less observed in the inner layer of molecular layer of DG and the layers outside of SLM in the CAs. In contrast, ions corresponding to C18-GD1 were detected in the whole region of the hippocampal formation, but the signals were weak in the DG-ML and the SLM. Ions corresponding to C18-GM1 were also detected in region rich in the myelinated axon. We also performed IMS of a horizontal brain section (Figure S2) (40 µm raster) and observed clear accumulation of C-20 gangliosides in the entorhinal cortex (EC) and the regions including projections from the EC both to the DG-ML and to the SLM of the hippocampus.

### Confirmation of IMS results by MS of methyl-esterified gangliosides

MALDI-MS of sialic acid-containing oligosaccharides should be performed with caution because of the preferential loss of sialic acid during mass spectrometry[28], [29]. To evaluate the degree of sialic acid loss in the experimental system used, we performed mass spectrometry of authentic samples of GM1, GD1, and GT1 in the presence of sodium and potassium at physiological concentrations, at same laser power and detector sensitivity used in the IMS experiments (Figure S3). We found that GD1 and GT1 preferentially formed sodium/potassium adduct ions under presence of salts, and that reduced the sialic-acid dissociation though GD1 produced certain amount of ions at *m/z* 1544 and 1572, which lost one sialic-acid. On the other hand, the presence of salts efficiently suppressed the loss of sialic acid from GT1. In authentic GM1 samples, there was almost no sialic-acid dissociation.

**Figure 4:**
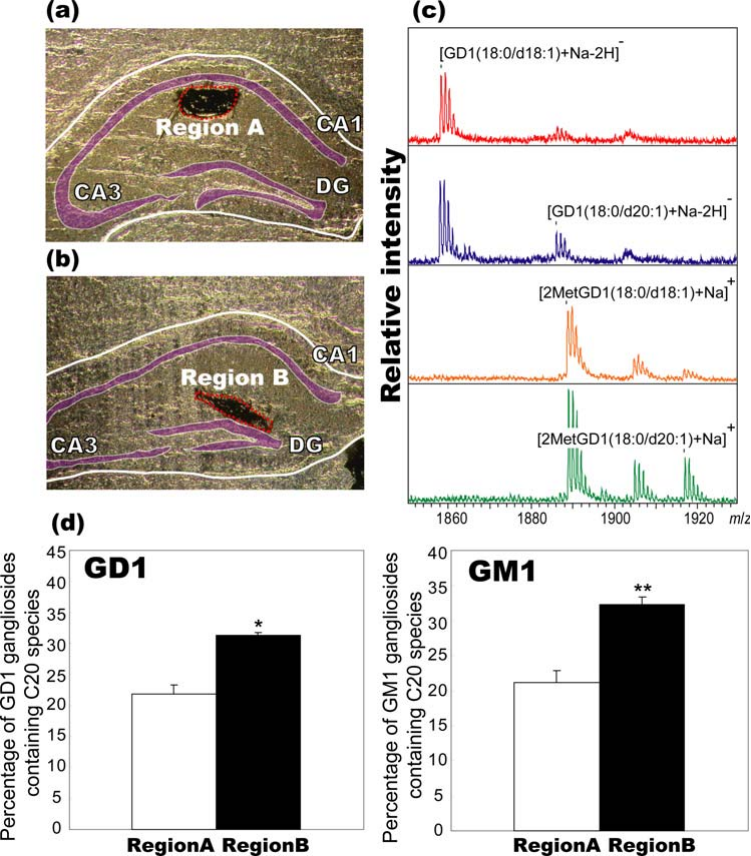
Localization of C20-sphingosine-containing gangliosides was confirmed by MS of extracted and methyl-esterified gangliosides. To determine the percentage of GM1/GD1 gangliosides containing the C20-species in different regions without allowing sialic acid dissociation during MS measurement, we extracted gangliosides from the dendritic region of the SR (region A, (a)) and the ML/SLM (region B, (b)). They were derivatized to methyl-esterified gangliosides. From the result of MS of underivatized gangliosides and methyl-esterified gangliosides (c), the percentage of GM1/GD1 gangliosides containing the C20-species were calculated (d). Three different mouse brain sections were used, and the data were expressed as mean±S.D. * and ** indicate P<0.05 and P<0.005, respectively, Student's *t*-test.

Thus, to confirm the IMS results, we extracted gangliosides from the regions of interest, i.e., the stratum radiatum (SR) (region A) and ML/SLM region (region B) in the mouse hippocampal formation (Figure 4). We then derivatized them to the methyl-esterified gangliosides. While underivatized GD1 and GT1 exhibited significant loss of sialic acid due to dissociation by in-source or post-source decay[28]–[30], such dissociation was suppressed by methyl esterification[31], enabling the detection of their fully sialylated molecules as dominant peaks. The results of MALDI-MS analysis of methyl-esterified GM1 and GD1 showed that the C20 molecular species was present in approximately 21% of the total GM1 gangliosides in region A and 32% of those in region B. Further, 21% and 34% of the GD1 gangliosides in region A and B, respectively, contained the C20 molecular species. Taken together, these results confirmed the accumulation of the C20 species in both GD1 and GM1 gangliosides in the ML and SLM.

### Changes in the distribution of ganglioside molecular species during development

**Figure 5:**
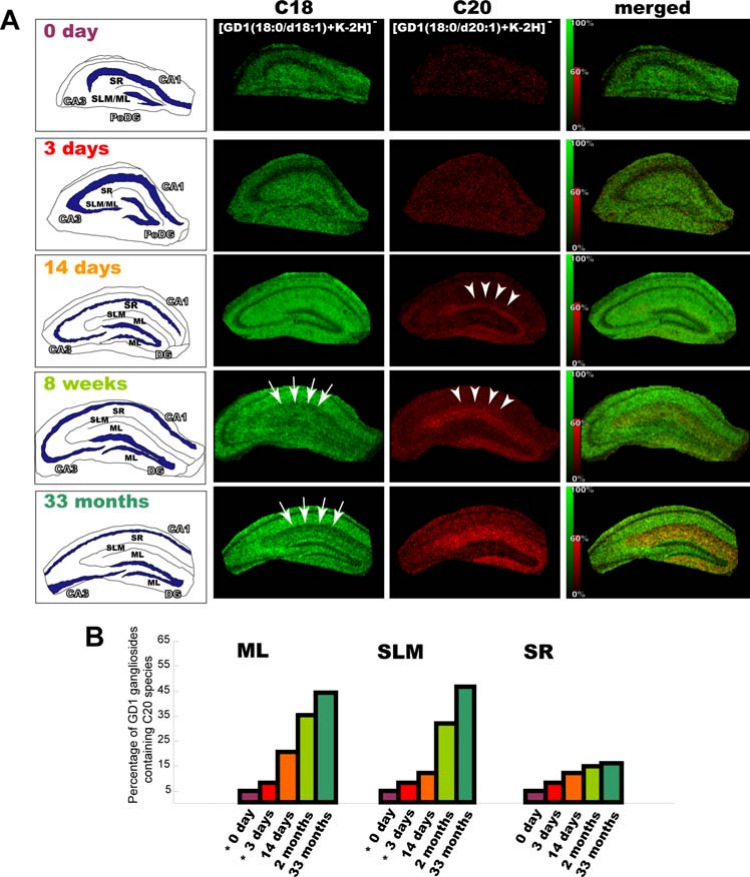
Development- and aging-related accumulation of C20-GD1 in the ML and SLM of the hippocampal formation. We visualized the ion corresponding to GD1 (m/z 1874 and 1902) in the mouse hippocampus at the indicated time points (P0, P3, P14, 1 month, and 33 months). For each time point, intensity scale of C20-GD1 is normalized in order that the brightest pixels of C20-GD1 have 60% of the maximal C18-GD1 intensity value. In the P14 mouse hippocampus, C20-GD1 was concentrated in the narrow area of DG-SMm and began to spread over the medial edge of the region (arrow heads). In contrast, the concentration of the C-18 species decreased in the ML/SLM with aging (arrows). Quantification result of C20-GD1 on the total GD1 signal in the ML, SLM and SR region has also been shown (B). *; At P0 and P3, we could not distinguish between the ML and SLM area; therefore, values obtained from the region corresponding to ML/SLM have been used for both the regions in the graph.

To date, several articles have reported development- and aging-related increase in the C20-ganglioside content[15]–[17], and we think that it is important to know both when and where C20-gangliosides accumulate. In order to identify and characterize gangliosides in developing and aged hippocampal formations, we performed IMS of the mouse hippocampal formation at 0, 3, and 14 postnatal days, 8 postnatal weeks, and 33 postnatal months. Figure 5 shows the IMS results for ions at *m/z* 1858 and 1902 and demonstrates that the area with high C20-GD1 content increased with neurodevelopment. On postnatal days 0 and 3, significant signals (S/N>1.0) derived from C20-GD1 were detected from only a few data points in the entire hippocampal formation. On postnatal day 14, C20-GD1 signals were concentrated in the narrow area of the DG-ML and began to be observed over the medial edge of the region, which corresponds to the terminal area of the projections from the lateral entorhinal area (Figure 5A, arrow heads)[32]. At 8 postnatal months, the signals were observed from a wide area (ML/SLM), which corresponds to the terminal area of the projections both from the lateral and medial entorhinal area[32]. Furthermore, in aged hippocampal formations, the accumulation was clearly increased. Figure 5B shows the percentage of GD1 gangliosides containing the C20-species in the different regions. It demonstrates the development- and aging-related increase in C20-GD1 content in the ML and SLM, but no obvious increase in other regions in the hippocampal formation. In contrast, the C18-GD1 content decreased in the ML/SLM with aging (Figure 5A, arrows).

## Discussion

As a next step of the previous study which characterized the distinct composition of ganglioside molecular species between axons/dendrites and soma of neuron *in vitro* [33], in the present study, we demonstrated that gangliosides with differences in their ceramide moieties showed distinct distribution patterns in the mouse brain, especially in the hippocampal formation *in vivo*.

In the direct analysis of gangliosides using MALDI-MS, the mass spectra showed distinct mass peaks for ganglioside molecular species with different oligosaccharide/ceramide moieties (Figure 2), which enabled the visualization of the distribution of individual molecular species by IMS (Figure 3). The characteristic of gangliosides specific to the CNS is the structure of their LCB, i.e., the presence of 18 or 20 carbons; further, C20-gangliosides are found only in the CNS[12]–[14]. Antibodies to the oligosaccharide moieties of gangliosides are used to visualize the distribution of gangliosides with different oligosaccharide moieties; however, antibodies cannot distinguish between the C18 and C20 molecular species. To date, no other method has achieved differential visualization of such molecular species.

MALDI-MS should be performed with caution when used for the detection of oligosaccharide moieties of gangliosides because previous MS studies of gangliosides have demonstrated that sialic acid residues tend to be lost[28]–[30]. Thus, it is necessary to evaluate the degree of sialic acid dissociation in the experimental system described here. We performed MS of authentic samples of GM1, GD1, and GT1 gangliosides, which account for 80% of the total brain gangliosides[6]. From the results, we deduced that the ion signals at *m/z* 1544 and 1572 correspond to ions originating from both GM1 and GD1, but not GT1. In contrast, the ions at *m/z* 1874 and 1902 contain almost no GT1-derived signals and originated predominantly from only GD1(Figure S3).

Based on these results, we analyzed the distribution patterns of the C20 species. The IMS results revealed that both C20-GM1 and C20-GD1 are selectively localized in the outer two-thirds of the DG-ML, among all brain regions (Figure 3B). The ion signal at *m/z* 1572 (C20-GM1/GD1) showed more concentrated pattern than that at *m/z* 1902(C20-GD1); this indicates that GM1 has a higher C20 content than GD1 in these regions. Moreover, this trend was confirmed by MS of methyl-esterified gangliosides after extraction from the tissue section (Figure 4)

Because most of the afferent nerves from the EC terminate in the SLM/ML in DG, C20-GM1 and GD1 are suggested to be most concentrated in the axon and the axon terminals of the neurons from the EC[32]. Furthermore, in the horizontal sections, C20-GM1/GD1 were localized in the lateral and medial parts of the EC area and the region including the projections (a medial and lateral perforant path) to the DG and the area in which they terminated[34]. These results suggest that EC neurons selectively express the C20 species. We deduce that for other gangliosides, in particular precursor-gangliosides to biosyntheses GM1 and GD1, namely GM2, GD2, GM3, and GD3, the C20-species of them are also localized in these regions. Although we could not detect a sufficient number of ions of these gangliosides, presumably because they are present in considerably smaller amounts than GM1 and GD1[6], this is an interesting topic for further study.

Moreover, observation of the concentration of C20 species throughout development suggests that the appearance of extensive C20-GM1/GD1 distribution in the DG-SMm corresponds to the period of rapid synapse formation, dendritic outgrowth, and glial proliferation in this region. Taken together, this C20-GM1/GD1 distribution and concentration may reflect the functional maturation of the EC-hippocampus neural pathway, which possibly progresses first from the LEA and then from the MEA area (Figure 5). Indeed, it is known that EC lesions induce changes in the ganglioside content in the hippocampus DG-ML[35]. Moreover, it is known that animals with EC lesions show behavioral deficits, and ganglioside administration accelerates the recovery of the impaired functions[34], [36], [37]. The present findings suggest that such ganglioside treatments have effects that are possibly dependent on the type of molecular species they contain. Furthermore, the IMS results suggest that aging-related increase in the C20-GM1/GD1 content, which has also been proven by the biochemical data obtained in studies using HPLC[11], [16], [17], [38], selectively occurred in the DG-ML/SLM region in the hippocampal formation. Since C20-sphingosine is more effective in reducing membrane fluidity than the C18 species, this age-dependent accumulation of C20-GM1 can lead to altered properties of the cell membrane. Considering the age-dependent accumulation and the selective distribution of the C20 species in the EC and its projections, where selective degradation of neurons is observed in early stages of Alzheimer diseases[39], this accumulation may increase the risk for the age-dependent neurological diseases such as Alzheimer disease.

Finally, in this study, we successfully characterized the location of age-dependent C20-GD1 accumulation (Figure 5) besides that previous studies have established this phenomenon with highly quantitative methods in brain lysate[11]. However, one should bear mind that IMS is developing method especially for quantitative analysis because of nature of MALDI, in which ionization efficiency of analyte is easily affected by number of factors such as crystallization condition of matrix and extraction efficiency of analyte from tissues [23]. We consider that established-quantitative methods such as HPLC are effective to complement its quantitative aspect of MALDI-IMS.

## Materials and Methods

### Chemicals

Methanol, trifluoroacetic acid (TFA) and methyl iodide were purchased from Wako Chemical (Tokyo, Japan). Calibration standard peptide and 2,5-dihydroxybenzoic acid (DHB) were purchased from Bruker Daltonics (Leipzig, Germany).

### Section Preparation

We used the brains of male C57BL/6Cr mice and at the indicated time point (0, 3, and 14 postnatal days, 8 postnatal weeks, and 33 postnatal months), they were sacrificed. The extirpated tissue blocks were immediately frozen in powdered dry ice and stored at −80°C until use. The frozen sections were sliced at −16°C with a cryostat (Leica CM 3050) at a thickness of 5 µm according to the previous reports [40], [41]. To fix each tissue block, an optimum cutting temperature (OCT) polymer was used. When the sections were sliced, the cutting block was not embedded in OCT since any residual polymer on the tissue slices might have degrade mass spectra[41]. Frozen sections were thaw-mounted on indium-tin-oxide (ITO)-coated glass slides (Bruker Daltonics) and ITO-coated sheets (Tobi Co., Ltd., Kyoto, Japan). ITO-coated glass slide was used for the measurement using TOF/TOF instrument and ITO-coated sheet was used for quadrupole ion trap (QIT)-TOF instrument. For matrix, we used a DHB solution (50 mg/mL; 70% methanol, 0.1% TFA) because this matrix minimizes the loss of sialic acid and carbon dioxide from gangliosides[28]. The matrix solution was uniformly sprayed over the tissue surface using a 0.2 mm nozzle caliber air-brush (Procon Boy FWA Platinum; Mr. Hobby, Tokyo, Japan). In this study, the distance between the brush's nozzle tip and the tissue surface was kept at 15 cm and the spraying period was fixed at 3 minutes. All experiments with mice were conducted using protocols approved by the Animal Care and Use Committee of the Mitsubishi Kagaku Institute of Life Sciences.

### Tandem Mass Spectrometry

For the MS^n^ analysis, we used a QIT-TOF mass spectrometer (AXIMA-QIT; Shimadzu, Kyoto, Japan). The MS^n^ analysis was performed directly on the hippocampus area of the mouse brain sections. Acquisition was performed in the “mid-mass range” mode (*m/z* 750–2000) at a stage voltage of −18 V in the negative-ion detection mode. In the MS^n^ analysis, the conditions for data acquisition (i.e., laser power, collision energy, and the number of laser irradiations) were changed in order to obtain product ion mass spectra with peaks that have high intensity and a high signal-to-noise ratio. The calibration was performed using an external calibration method.

### Protocols of IMS

IMS were performed using a MALDI time-of-flight (TOF)/TOF-type instrument (Ultraflex 2 TOF/TOF; Bruker Daltonics). This instrument was equipped with a 355 nm Nd:YAG laser. The data were acquired in the negative-reflectron mode under an accelerating potential of 20 kV by using an external calibration method. In this analysis, signals between *m*/*z* 800 to 2500 were collected. The raster scan on the tissue surface was performed automatically by FlexControl and Fleximaging 2.0 software (Bruker Daltonics). The number of laser irradiations was 100 shots in each spot. Image reconstruction was performed using FlexImaging 2.0 software.

### Data processing

In the IMS results, the variation in the ionization efficiency, which is caused by the heterogeneous distribution of matrix crystals and their sublimation during measurement, was eliminated for each data point by equalizing the total ion current of each mass spectra, using the “Normalize Spectra” function of FlexImaging 2.0 software. In addition, in IMS of developing hippocampus formation (Figure 5), for each time point, intensity scale of C20-GD1 is normalized in order that the brightest pixels of C20-GD1 have 60% of the maximal C18-GD1 intensity value using the “Edit Mass Filter Parametrs” function of FlexImaging 2.0 software.

For calculation C20-ganglioside percentage (Figure 5), we used the most intense ion peak derived from GM1 and GD1, namely [GM1-H] ^−^ and [GD1+K-2H]^−^ , and intensities of these peaks in the summed spectra from each hippocampal region (at least 1300 spectra were summed for one region) were used for the calculation. For spectrum summation, Flex Analysis 3.0 software was used (Bruker Daltonics).

### Analysis of Methyl-esterification of gangliosides

Tissues of the interested regions were dissected using an injection needle (Terumo 22G needle; Terumo Corporation, Tokyo, Japan) under stereo-microscopic observation and immediately immersed in 20 µl of methanol. After vortexing, the solution was centrifuged and the supernatants were added to 6 µl of methyl iodide. The reaction was performed for 3 h at room temperature. Gangliosides in the reaction mixture were eluted from a PepClean C18 spin column (Thermo Fisher Scientific, Kanagawa, Japan), according to the procedure described by S. Handa and K. Nakamura [42]. Mass spectrometry was performed with TOF/TOF instrument using DHB as matrix (5 mg/mL; 50% methanol, 0.1% TFA), on the steel target plate (MTP 384 target plate ground steel; Bruker Daltonics).

## Supporting Information

Figure S1 Structures of ganglioside molecular species containing C18-LCB and C20-LCB. C20 species has 2 more carbons in their LCB moiety than C18 species (arrow).
(0.48 MB TIF)

Figure S2 C20 gangliosides were concentrated in the dendritic region of hippocampal granule neurons. A. Low-resolution MSI (40 µm raster) was performed to gain an overview of ganglioside expression in the horizontal section of mouse brain. For ions corresponding to the GD1 molecular species, we visualized the ion distribution of the potassium complex, i.e., the ions at *m/z* 1874 and *m/z* 1902, which correspond to the [M+K-H]- form of C18- and C20-GD1, respectively. For those corresponding to GM1, the ions at *m/z* 1544 and *m/z* 1572, which correspond to C18-spingosine- and C20-sphingosine-containing GM1 species, respectively, are shown. B. To show the projections from the EC to the DG, an optical image of successive sections stained by the KB method has been presented.
(6.37 MB TIF)

Figure S3 Formation of sodium/potassium complex ion suppressed loss of sialic-acid in GD1 and GT1 ganglioside. A. Summary of the results of the MALDI-MS experiments performed using authentic samples of GM1 (a), GD1(b), and GT1 (c), which were analyzed in the presence/absence of sodium and potassium at physiological concentrations. (d) The spectra obtained from the mouse hippocampal formation. B. Gangliosides were detected without (white bar), and with 1 (black bar), and 2 (gray bar) sialic-acid dissociated forms
(1.65 MB TIF)
