# Supplementary figures and images for: Imaging Mass Spectrometry Technology and Application on Ganglioside Study; Visualization of Age-Dependent Accumulation of C20-Ganglioside Molecular Species in the Mouse Hippocampus
Source: PLoS One. 2008 Sep 18;3(9):e3232. doi: 10.1371/journal.pone.0003232 (PMC2532745; doi:10.1371/journal.pone.0003232)

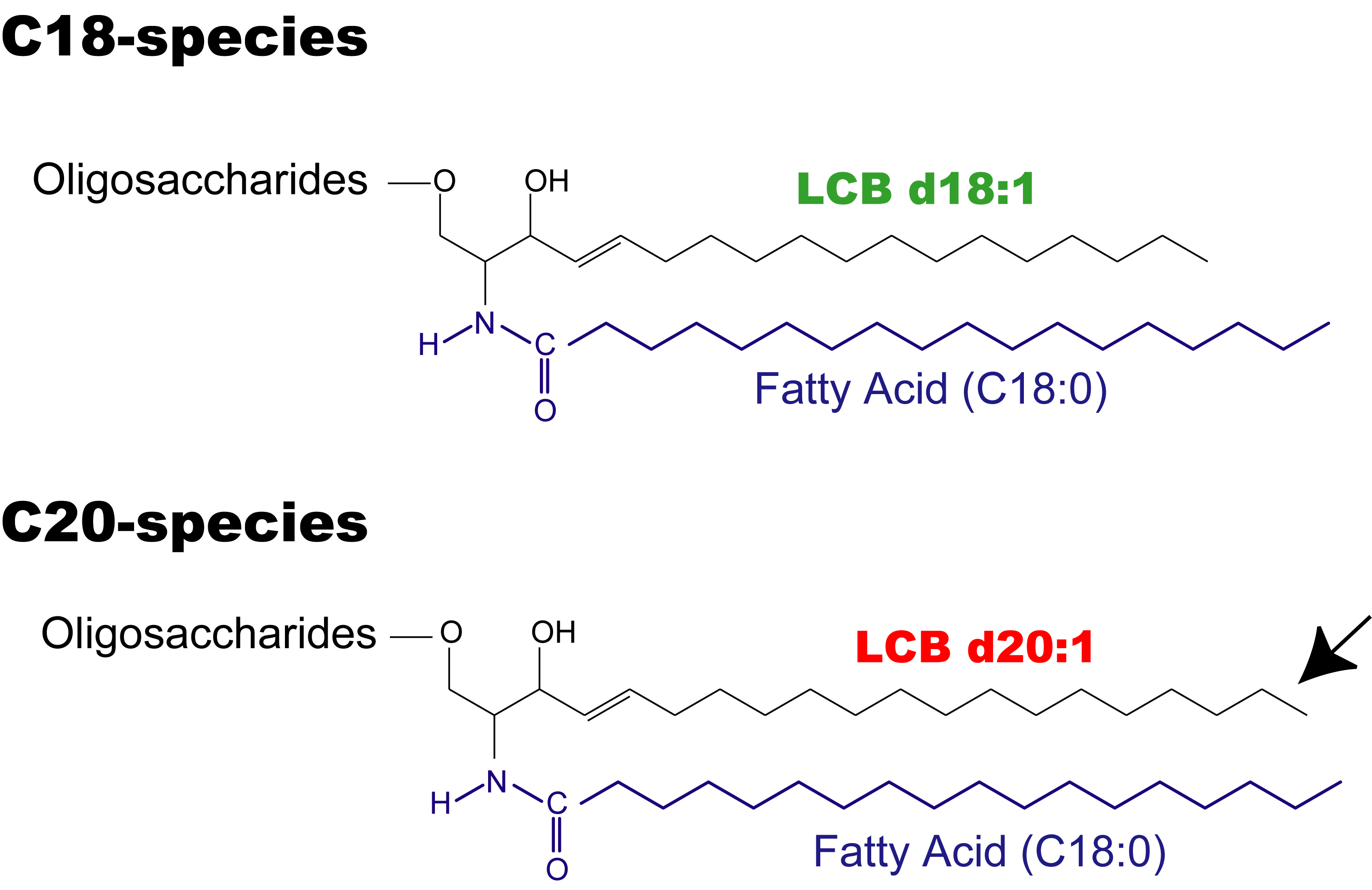

Supplement: Figure S1 — Structures of ganglioside molecular species containing C18-LCB and C20-LCB. C20 species has 2 more carbons in their LCB moiety than C18 species (arrow). (0.48 MB TIF) [file pone.0003232.s001.tif]

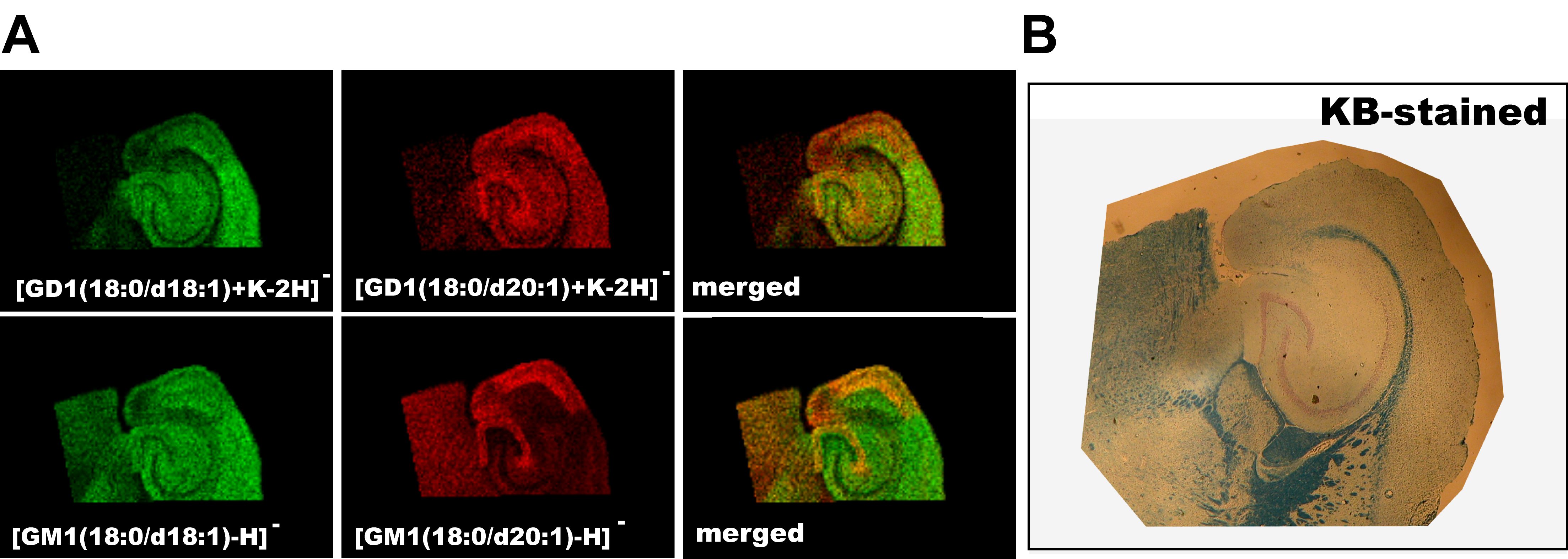

Supplement: Figure S2 — C20 gangliosides were concentrated in the dendritic region of hippocampal granule neurons. A. Low-resolution MSI (40 µm raster) was performed to gain an overview of ganglioside expression in the horizontal section of mouse brain. For ions corresponding to the GD1 molecular species, we visualized the ion distribution of the potassium complex, i.e., the ions at m/z 1874 and m/z 1902, which correspond to the [M+K-H]- form of C18- and C20-GD1, respectively. For those corresponding to GM1, the ions at m/z 1544 and m/z 1572, which correspond to C18-spingosine- and C20-sphingosine-containing GM1 species, respectively, are shown. B. To show the projections from the EC to the DG, an optical image of successive sections stained by the KB method has been presented. (6.37 MB TIF) [file pone.0003232.s002.tif]

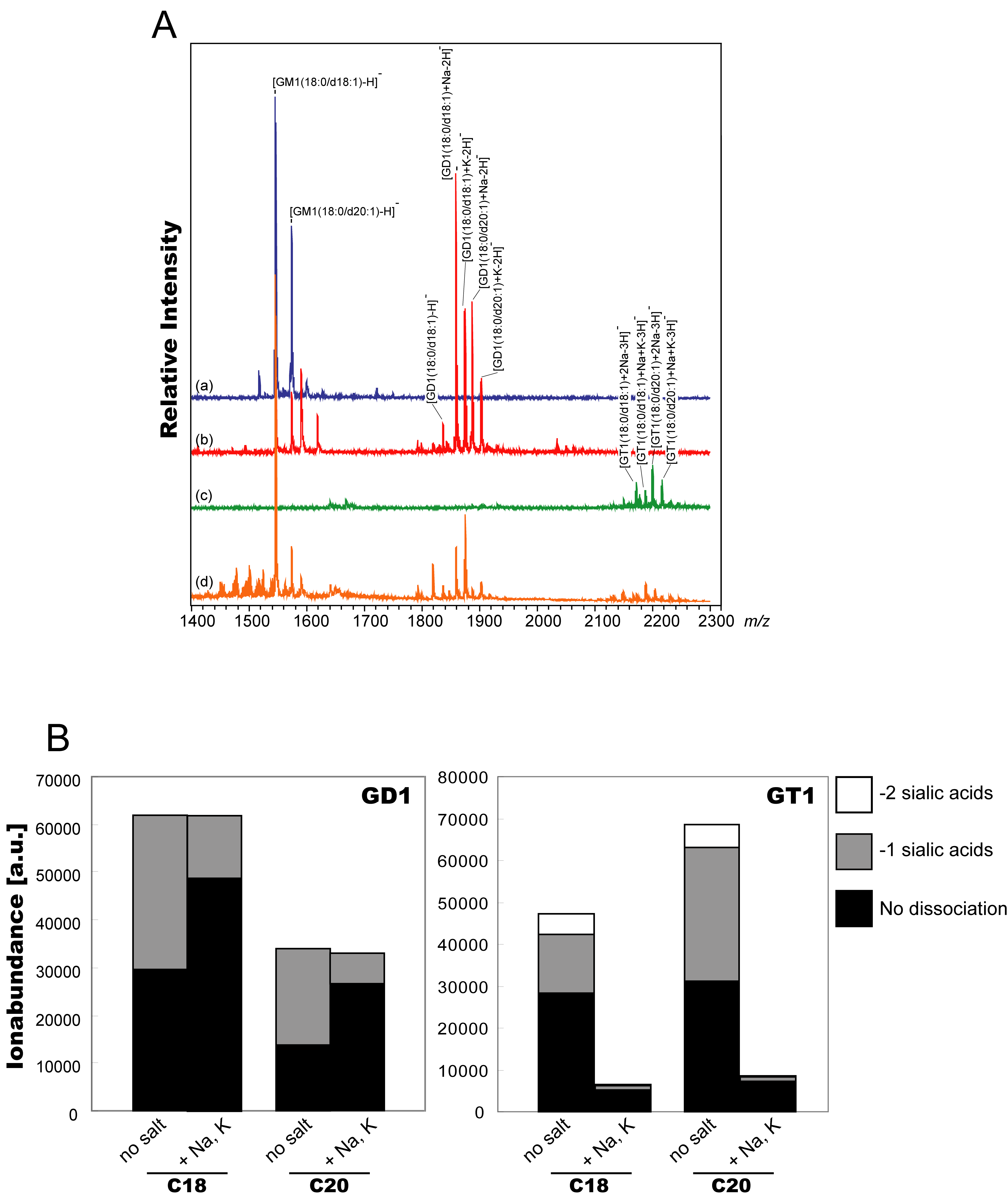

Supplement: Figure S3 — Formation of sodium/potassium complex ion suppressed loss of sialic-acid in GD1 and GT1 ganglioside. A. Summary of the results of the MALDI-MS experiments performed using authentic samples of GM1 (a), GD1(b), and GT1 (c), which were analyzed in the presence/absence of sodium and potassium at physiological concentrations. (d) The spectra obtained from the mouse hippocampal formation. B. Gangliosides were detected without (white bar), and with 1 (black bar), and 2 (gray bar) sialic-acid dissociated forms (1.65 MB TIF) [file pone.0003232.s003.tif]
